# Usability and Acceptance of a Social Robot in People With Multiple Sclerosis: Comparative Cross-Sectional Study

**DOI:** 10.2196/94068

**Published:** 2026-10-21

**Authors:** Jessica Podda, Alice Bollini, Alessia Susini, Domenico Presenza, Keven T Kearney, Ester Cecchella, Erica Grange, Mario Alberto Battaglia, Andrea Tacchino, Giampaolo Brichetto

**Affiliations:** 1 neuroBRITE Research Center, Italian Multiple Sclerosis Foundation, Via Operai 40, Genoa, 16149, Italy, +39 010 2713306; 2 R&I Lab, Engineering Ingegneria Informatica S.p.A, Rome, Lazio, Italy; 3 Department of Informatics, Bioengineering, Robotics and Systems Engineering (DIBRIS), University of Genoa, Genoa, Italy; 4 Department of Life Science, University of Siena, Siena, Italy; 5 Italian Multiple Sclerosis Foundation, Genoa, Italy; 6 AISM Rehabilitation Service, Italian Multiple Sclerosis Society, Genoa, Italy

**Keywords:** socially assistive robotics, multiple sclerosis, usability, technology acceptance, anthropomorphism, human-robot interaction, natural language processing, Pepper

## Abstract

**Background:**

Socially assistive robotics (SAR) is an emerging field integrating robotics, medicine, cognitive and social sciences, and neuroscience. Its potential in multiple sclerosis (MS) remains largely unexplored, although MS affects sensory-motor, cognitive, and psychological domains where SAR could offer substantial benefits.

**Objective:**

This study aimed to evaluate the usability of a humanoid robot in people with MS, compare it with age-matched healthy individuals (HIs), and explore usability predictors. Automated written-language analysis was tested as a complementary approach.

**Methods:**

In this 2025 cross-sectional study at the Italian MS Foundation laboratory, 20 people with MS (mean age 51.4, SD 11.0 years; n=13, 65% female; mean education 13.0, SD 3.4 years) and 20 age-matched HIs (mean age 47.3, SD 10.1 years; n=12, 60% female; mean education 14.8, SD 2.2 years) completed 4 predefined tasks with the robot Pepper. Usability was assessed using the System Usability Scale (SUS). Attitudes toward robots, robot-related factors, and task-related effort were measured with the General Attitudes Towards Robots Scale (GAToRS), Godspeed questionnaire, and NASA-TLX, respectively. Psychological variables (eg, personality traits from the Italian Ten-Item Personality Inventory [I-TIPI]) and clinical characteristics for people with MS (eg, cognitive functions via the app DIGICOG-MS, upper-limb difficulties with the Arm Function in Multiple Sclerosis [AMSQ]) were collected. Written responses to open-ended questions were analyzed using natural language processing (NLP).

**Results:**

SUS scores exceeded the standard cut-off in both groups, with no between-group differences (people with MS: mean 81.00, SD 12.31; HIs: mean 77.75, SD 11.24; *r*=0.14, 95% CI 0.01-0.42; *P*=.39), indicating high usability. People with MS reported more negative attitudes toward robots at the societal level (GAToRS S–: *r*=0.36, 95% CI 0.08-0.63; *P*=.02) and judged Pepper as more anthropomorphic (*r*=0.42, 95% CI 0.15-0.67; *P*=.007) and intelligent (*r*=0.37, 95% CI 0.09-0.61; *P*=.02) than HIs. Anthropomorphism was strongly associated with visuospatial memory (DIGICOG-MS: ρ=–0.632, 95% CI –0.84 to –0.26; *P*=.003) and relatively strong with both verbal memory (DIGICOG-MS: ρ=–0.504, 95% CI –0.77 to –0.08]; *P*=.02) and agreeableness (I-TIPI: ρ=0.491, 95% CI 0.06-0.77; *P*=.03). Exploratory forward stepwise regression showed that conscientiousness (I-TIPI: *B*=2.064, 95% CI 0.679-3.449), negative personal attitudes toward robots (GAToRS P–: *B*=0.169, 95% CI 0.033-0.306), and perceived upper-limb difficulties (AMSQ: *B*=0.169, 95% CI 0.033-0.306) explained 64% of SUS variance (adjusted *R*²=0.568); task-related frustration (NASA-TLX: *B*=–1.497, 95% CI –2.276 to –0.719) explained 48% (adjusted *R*²=0.451). NLP analyses revealed no between-group differences in lexical productivity or sentiment-lexicon coverage.

**Conclusions:**

This study provides first preliminary evidence that people with MS perceive a social robot as highly usable, comparably to HIs, suggesting MS-related challenges may not hinder human-robot interaction. Psychological factors, prior attitudes toward robots, and clinical characteristics were associated with user experience in the MS group. Future research should explore whether integrating these measures with language-based approaches informs tailored interventions in MS.

## Introduction

Socially assistive robotics (SAR) is a growing interdisciplinary research field aiming at supporting people in different social situations through robot-based solutions [1]. This area integrates robotics, medicine, social and cognitive sciences, and neuroscience. Social robots are used in various domains, such as health care, education, business, industry, and culture [2], where interaction between individuals is essential. One of the main challenges of human-robot interaction applied to different research areas, such as physical and cognitive rehabilitation or emotional support, lies in ensuring a fluid and effective interplay so that users have the subjective impression that they are interacting with an intelligent agent. In this context, achieving a positive user experience (UX) is crucial, as it directly influences engagement, acceptance, and the overall impact of SAR on human life [2]. SAR has demonstrated its value to promote people’s well-being by creating new avenues to access services and resources for mental health in real-world contexts [3], offering numerous possibilities for the innovation and implementation of robots in education, medicine, rehabilitation, and psychotherapy, improving treatment compliance in children with specific clinical conditions such as autism spectrum disorder [4-8] and in reducing anxiety among older adults, with subsequent improvements in their quality of life [9]. As indicated in a recent review [10], SAR has been used with promising success specifically in supporting everyday life, providing interaction, and facilitating cognitive and physical training in the older population. Despite these advances, the use of SAR in other neurological populations [11-13], such as people with multiple sclerosis (MS), remains largely unexplored. This is surprising given that MS is characterized by a heterogeneous combination of sensory-motor impairments, cognitive deficits, and psychological symptoms, all domains in which SAR could potentially offer substantial benefits [14,15]. The impact of MS extends into work roles, economic status, and social relationships. It is reasonable to surmise, therefore, that MS and its manifestations make those living with this disease vulnerable to the experience of loneliness and social isolation [16,17]. In this context, SAR may offer a promising avenue not only for physical and cognitive rehabilitation but also for addressing the social and emotional needs of people with MS—a gap that has received little attention in the existing literature. However, before implementing such interventions, it is crucial to understand how people with MS perceive and interact with social robots. To date, there is no empirical evidence on how this clinical population perceives key social dimensions of human-robot interaction, such as robot anthropomorphism. Without this foundational knowledge, deploying SAR in MS rehabilitation risks being ineffective or misaligned with people’s needs.

Based on these premises, the aims of this study were to (1) evaluate the usability of humanoid robot Pepper (SoftBank Robotics) in people with MS; (2) investigate any differences with an age-matched group of healthy individuals (HIs) in both perceived usability and task-related effort; and (3) exploratorily examine potential predictors of usability, including psychological variables, prior attitudes toward robots, and clinical characteristics. Furthermore, as a complementary component of the study, we applied natural language processing (NLP) techniques to participants’ written responses to open-ended questions with the aim of examining whether automated language analysis could serve as a proxy for evaluating user appreciation of the social robot alongside the primary quantitative measures. This analysis was not intended as a substitute for qualitative inquiry, but rather as a preliminary, hypothesis-generating exploration of the linguistic correlates of UX in this population.

## Methods

### Study Population

This cross-sectional study was conducted in November 2025 at the laboratory of the Fondazione Italiana Sclerosi Multipla (FISM) in Genoa (Italy). The study sample consisted of people with MS, followed as outpatients at the Italian Multiple Sclerosis Society (AISM) Rehabilitation Service of Liguria (Genoa, Italy). Potential participants were identified by the AISM clinical staff and approached directly during routine clinical appointments or contacted by telephone by the FISM Secretariat. All individuals contacted agreed to participate, except for 1 individual who expressed willingness but was unable to attend due to work commitments. Eligibility criteria included an age of 18 years and older; a confirmed MS diagnosis [18]; any disease course (relapsing-remitting, secondary progressive, primary progressive MS); the absence of relapses in the last 3 months; an Expanded Disability Status Scale (EDSS) [19] of 6.5 or below; and adequate visual, hearing, and motor capabilities to complete the experimental tasks. Exclusion criteria were a Montreal Cognitive Assessment score of 18 or below [20], neurological and major psychiatric illness, past serious head trauma, and alcohol or drug abuse. Inclusion criteria were intentionally broad to reflect the heterogeneity of the real-world MS population; eligible individuals were first identified by the clinical staff in collaboration with the research team, ensuring that only those meeting all eligibility criteria and able to safely complete the experimental tasks were approached. A healthy control group was included to provide a reference for comparison, allowing us to determine whether people with MS respond to the robot differently from HIs, and to identify usability patterns and attitudes that may be specific to the MS population rather than common to all users. HIs were recruited on a voluntary basis among AISM employers through institutional email distribution, matched by age, and were required to have no history of neurological or psychiatric diseases. All participants provided written informed consent prior to enrollment.

### The Social Robot and Software Used in the Experiment

The device used in this study was the humanoid social robot Pepper. Pepper is designed to interact with people and is capable of autonomous navigation, face recognition, scripted dialogue, and adopting life-like human poses, such as performing arm and hand gestures. In this study, Pepper was programmed with a small and simple set of behaviors: greeting participants, providing instructions for each experimental task, and guiding participants through the tasks themselves, as detailed in the next section (Figure 1). The software controlling the robot’s behaviors in the study was developed by Engineering Ingegneria Informatica S.p.A. (EII) as part of their long-term Socially Assistive Robotic Solution for Ambient (SARA)–assisted living, a complex distributed home automation and robotics system providing health monitoring and (socially interactive) assistance in daily living tasks to older adults and their caregivers at home, with the aim of prolonging their autonomy and delaying their institutionalization. Following [21], the SARA has the ambitious goal to develop robots that provide physical assistance, for example, rehabilitation robots, wheelchair robots, mobility aids, manipulator arms), and are able to engage humans in interaction, for example, from [22] engaging in high-level dialog, using and understanding natural cues (gaze, gesture, etc), expressing/perceiving emotions, and establishing and maintaining social relationships (among other competencies).

**Figure 1.**
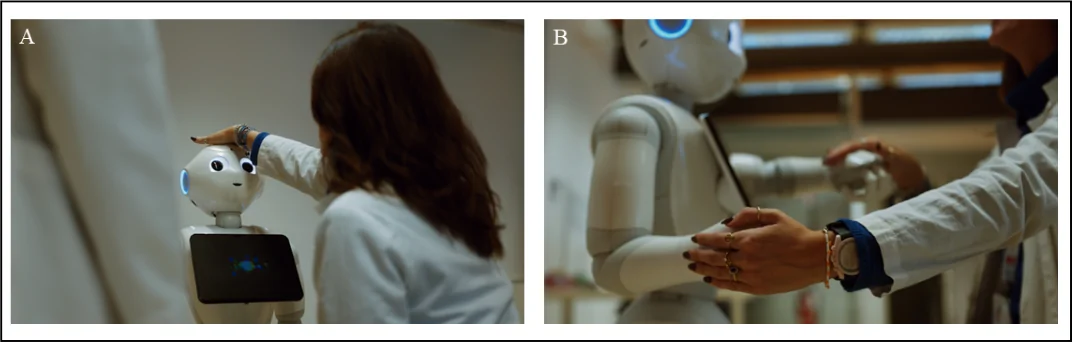
Participant-robot interaction with the humanoid social robot Pepper (SoftBank Robotics, Tokyo, Japan). (A) All interactions started with a head-touch gesture to activate the robot’s social engagement mode. (B) Keyframe from 1 experimental task, showing the reciprocal movement dynamics between the participant and the robot.

### Experimental Procedures: Human-Robot Interaction

Prior to the experimental sessions, a preliminary focus group was conducted with a multidisciplinary team, including clinicians with a high experience in MS (eg, psychologists, physiotherapists, occupational therapists) and engineers, to identify the most appropriate and meaningful tasks for the study. Insights from this focus group were used to select and refine task scenarios that were engaging and feasible for interaction with the robot. This collaborative approach ensured that the activities included in the experiment were ecologically valid and aligned with both abilities and practical considerations of people with MS for the robot’s implementation.

Each participant, in a structured setting, was invited to engage in 4 predefined interaction sequences with the robot (inspired by Tleubayev et al [23]), served to document engagement levels and usability perceptions of the robotic system, under the supervision of a trained health care professional (ie, a psychologist). The first 2 tasks were “static,” with the participant sitting in front of the robot, which remained docked (in a standing pose) on its recharging station throughout. Both tasks relied principally on the robot’s touch sensors and its upper body motion capabilities. The tasks were as follows:

- Task 1—Make me pose: The goal for the participant, seated in front of Pepper, was to make the robot adopt a target pose (demonstrated by the robot at the beginning of the interaction). Starting from a random initial pose, the robot moved into a new pose each time the participant touched the robot’s hands. The task stopped when the participant recognized that Pepper reached the target pose, indicated by the participant touching the robot’s head. The aim of this exercise was to stimulate the participant’s memory ability.
- Task 2—Keep in touch: The objective for the participant, again seated in front of Pepper, was to maintain physical contact between her/his hands and the robot’s hands while the robot executed a slow preplanned sequence of arm movements. The task stopped at the end of the sequence. The aim of this exercise was to stimulate participants’ focused attention while engaging in controlled upper body movements.

The remaining tasks were more dynamic, with both Pepper and the participant free to move around. The robot’s behavior in these tasks relied on its autonomous navigation capabilities and used proximity sensors (infrared, sonars, and lasers) embedded within its mobile base to detect the relative location of the participant. The final task also depended on the native visual localization functions of the robot. The 2 dynamic tasks were as follows:

- Task 3—Dance with me: The participant stood at 1 m or so in front of Pepper. The goal was to guide the robot through a short sequence of locomotive movements (forward, backward, and turning). The robot moved in response to changes in the relative positions of the participant and the robot—for example, if the participant stepped backward, Pepper moved forward—to maintain a constant relative distance and orientation. The aim of this exercise was to stimulate working memory and spatio-temporal orientation with the participant engaging in simple locomotion.
- Task 4—Drive me there: Once again the participant was standing, but now the objective was to guide the robot to a nearby location (a few meters away). The robot moved in response to the participant’s movements in the same way as in the previous Task 3. The aim of this exercise was to stimulate spatio-temporal orientation and planning abilities with the participant engaging in simple locomotion.

Each task was introduced and explained to the participant by the robot itself. During the task, the robot also offered occasional encouraging remarks and provided feedback on the participant’s progress, which were all accompanied with appropriate hand/arm and head gestures to favor communication and avoid ambiguity.

The experimental session lasted 45 minutes, comprising interaction with Pepper (20 min) and test and questionnaire administration (25 min). Figure 2 shows an overview of the 4 experimental tasks illustrating human-robot interaction.

**Figure 2.**
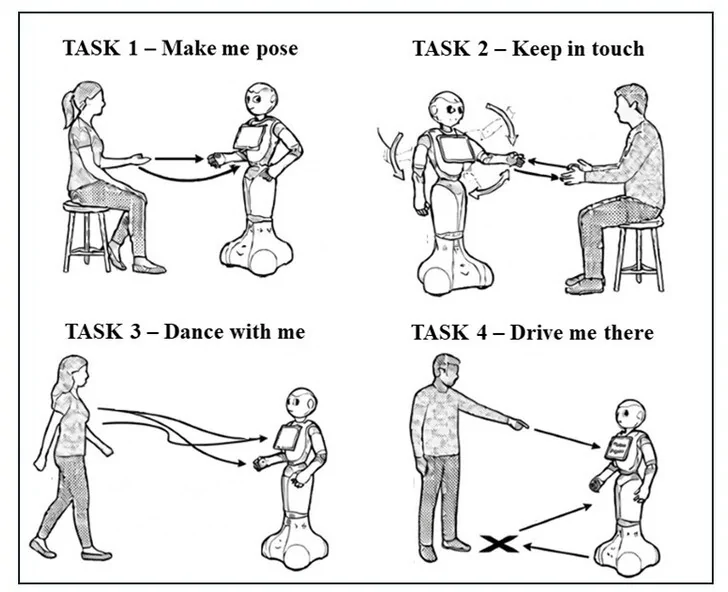
Overview of the 4 experimental tasks illustrating human-robot interaction. Participants performed 4 tasks with Pepper. In Task 1 (Make me pose), a seated participant guided the robot to a target pose via touch. In Task 2 (Keep in touch), a seated participant maintained continuous contact with the robot’s hands while it executed a sequence of movements. In Task 3 (Dance with me), a standing participant led the robot through locomotor movements using a “follow-the-leader” approach. In Task 4 (Drive me there), a standing participant guided the robot to a target location.

### Demographic and Clinical Outcomes

Sociodemographic data (ie, age, sex, educational level) were collected for all participants, while disease-related information (ie, MS course, disease duration, and disability level as indicated by EDSS) was gathered only for people with MS. Furthermore, MS cognitive functioning was tested using DIGICOG-MS, a mHealth app including digital tests that measure visuo-spatial and verbal memory, information processing speed, and semantic fluency [24]. Patient-reported outcomes (PROs), routinely used in MS clinical practice and well-established in this population, were administered to people with MS to assess upper-limb functionality (Arm Function in Multiple Sclerosis [AMSQ] [25]), perceived fatigue (Modified Fatigue Impact Scale [MFIS] [26,27]), perceived cognitive deficit (Multiple Sclerosis Neuropsychological Questionnaire [MSNQ] [28]), and impact of MS (29-item Multiple Sclerosis Impact Scale [MSIS 29] [29]). State and trait anxiety (State-Trait Anxiety Inventory [STAI] [30]), risk of depression (9-item Patient Health Questionnaire [PHQ-9] [31,32]), and personality traits (Italian Ten-Item Personality Inventory [I-TIPI] [33]) were collected for both people with MS and HIs. All questionnaires were administered in paper-and-pencil format, except for DIGICOG-MS, which was completed digitally. Participants completed all measures independently, with a psychologist available to clarify any questions. All instruments have been validated in Italian, with the exception of the MSIS-29, for which no formal Italian-language validation study has been published to date; however, its Italian translation is widely used in MS research internationally.

### Human-Robot Interaction Outcomes

Before starting the experiment, all participants completed the General Attitudes Towards Robots Scale (GAToRS) [34] to explore positive and negative attitudes toward robots at a personal and societal level. After the task, the following questionnaires were administered: System Usability Scale (SUS) [35] for overall usability (primary outcome); Godspeed questionnaire [36,37] for robot-related factors such as Anthropomorphism (ANTR), Animacy (ANIM), Likeability (LIKE), Perceived Intelligence (INT), or Perceived Safety (SAF) of robots; the National Aeronautics and Space Administration Task Load Index (NASA-TLX) [38] for mental and physical workload.

After the interaction with the robot, participants responded to three open-ended written questions: (1) “How would you describe your experience with the social robot during the activity?” (2) “What kind of feelings or thoughts did the interaction with the robot evoke?” (3) “Was there anything that struck you or surprised you about the way the robot interacted with you?”

### Statistical Analysis

Given the absence of prior SAR studies in people with MS, no formal a priori power analysis was feasible. The sample size of 20 per group was chosen in line with established practice in pilot SAR research, where small samples are commonly adopted when exploring novel populations or contexts [39,40]. Descriptive statistics including means, SDs, SEs, and medians with IQRs were calculated to summarize the data. Depending on the distribution of the data, parametric tests, nonparametric tests, or chi-square tests were used to compare demographic, psychological, and technological/robotic factors between people with MS and HIs, as appropriate. To explore factors associated with the group differences, exploratory bivariate correlations with clinical, psychological, and functional variables were run. Pearson or Spearman correlation analyses, as appropriate, were performed to examine associations between variables, with correlation coefficients interpreted as follows: 0.20-0.39 were interpreted as moderate, 0.40-0.59 as relatively strong, 0.60-0.79 as strong, and ≥0.80 as very strong correlation [41]. Effect sizes for Mann-Whitney *U* tests were reported as *r* (Z/√N) and interpreted as small (<0.30), medium (0.30‐0.50), or large (>0.50). Effect sizes for χ^2^ tests were reported as Cramér *V*. Given the lack of evidence on factors influencing the usability of social robots in people with MS and the small sample size (n=20), 2 exploratory stepwise linear regressions were conducted—one examining psychological factors, the other technological/robotic factors—to identify possible predictors, both using a forward selection procedure, entering all candidate predictors simultaneously and retaining only those meeting the p-to-enter threshold of .05 and p-to-remove threshold of .10. Particularly, the first stepwise multiple linear regression analysis was conducted to explore the extent to which personality traits (I-TIPI), attitudes toward robots (GAToRS), and other clinical variables such as perceived upper-limb difficulty (AMSQ), depression (PHQ-9), perceived cognitive abilities (MSNQ), impact of MS (MSIS), and perceived fatigue (MFIS) predicted SUS scores. The second analysis was run with workload as measured by NASA-TLX subdomains and Godspeed. Both *R*² and adjusted *R*² were reported to account for the number of predictors. Influential-case diagnostics were conducted for both regression models using Cook distance, with a threshold of 4/n=0.20. Prior to interpretation, model assumptions were verified through visual inspection. Normality of residuals was assessed using P-P plots, and homoscedasticity was examined through residual scatterplots, which revealed no systematic patterns, supporting the assumption of homoscedasticity in both models. Multicollinearity was evaluated using tolerance statistics and variance inflation factor (VIF) values. Given the exploratory nature of this study, no formal corrections for multiple comparisons were applied. While this increases the risk of type I error, applying corrections such as Bonferroni or FDR in exploratory analyses raises the risk of type II error and may obscure potentially meaningful patterns worthy of further investigation in confirmatory studies. Our analytical strategy aimed to capture the widest possible range of behavioral and clinical predictors, allowing for a more inclusive exploration of the factors shaping robot-assisted interaction in this clinical population.

In addition, participants provided short written responses to 3 open-ended questions about their experience interacting with the social robot. These responses were analyzed using a lightweight NLP pipeline aimed at extracting basic lexical and evaluative features. This analysis was conceived as exploratory and complementary to the primary quantitative outcomes, rather than as a stand-alone qualitative investigation.

Text preprocessing and lemmatization were conducted using the Italian language model in the spaCy NLP library [42]. Punctuation, numbers, and Italian stopwords were removed before analysis. Stopword lists were based on standard Italian Snowball resources [43] and were supplemented with additional context-specific terms related to the experimental setting to avoid artificially inflating lexical counts. The additional terms excluded were the following Italian words: *robot* (“robot”), *pepper* (“Pepper”), *esperienza* (“experience”), *interazione* (“interaction”), *attività* (“activity”), *esercizio* (“exercise”), *studio* (“study”), *compito* (“task”), *task* (“task”), *persona* (“person”), and *paziente* (“patient”). Lemmatized tokens were then used to compute basic lexical features, including total word count (NumWords) and the number of unique lemmas. To capture evaluative language, tokens were matched against the Sentix Italian sentiment lexicon [44,45]. For each participant, we calculated the number of tokens appearing in the sentiment lexicon (SentN). Because longer responses naturally increase the likelihood of lexicon matches, we derived an additional measure to estimate sentiment-lexicon coverage independently of response length. Specifically, SentN was regressed on total word count, and the residuals from this model were extracted. This residualized measure reflects whether a response contains more or fewer sentiment-lexicon matches than would be expected given its length. Responses to the 3 questions were concatenated to obtain a single text sample for each participant. The corpus therefore comprised 120 written responses from 40 participants (3 responses per participant). After preprocessing, 648 lemmatized tokens were retained for analysis, corresponding to a mean of 16.2 tokens per participant (SD 11.9; median 11.5; range 2‐50). Given the exploratory nature of the NLP component, no between-group test was formally prespecified. Both Welch 2-tailed *t* tests and Mann-Whitney *U* tests were conducted as complementary robustness checks. Within the MS group, exploratory associations between linguistic variables and UX measures (SUS and GAToRS) were examined using Kendall tau (*τ*), which is suitable for small samples and variables with tied ranks. To assess the sensitivity of the association between residualized sentiment-lexicon coverage and SUS to individual observations in the small MS sample, leave-one-participant-out and leave-two-participants-out analyses were additionally performed, reestimating the residualization model at each iteration. No formal multiplicity correction was prespecified for the exploratory NLP analyses. For the 4 reported NLP associations, Benjamini-Hochberg FDR-adjusted *P* values were calculated post hoc as a sensitivity analysis.

### Ethical Considerations

The study has been approved by the Regional Ethics Committee (229/2022 - id 12330), and participants provided their written consent to participate after reading information about the study in accordance with the revised Declaration of Helsinki. Data collected were stored in an anonymized format to properly protect the privacy and confidentiality of participants, ensuring that no individual can be identified from the data provided. Participants have been informed that data collection could be used only for research purposes.

## Results

### Sample Characteristics

Twenty people with MS and 20 HIs were enrolled and completed the study. As indicated by Mann-Whitney *U* tests, the groups did not differ in terms of demographic variables (all *P* values >.05), including sex distribution (*χ*²_₁_=0.107, *P*=.74, Cramér *V*=0.052; Table 1). Considering psychological variables, MS groups showed higher depressive symptoms compared to HIs (*P*<.001, *r*=0.70). This finding is consistent with evidence showing a higher prevalence of mood disturbances in people with MS compared to the healthy population [46]. No other differences have been observed between groups (all *P* values >.05). See Table 1 for a detailed summary of descriptive analysis of both groups considering demographic and psychological variables and Table 2 for clinical and disease-related information from people with MS.

**Table 1.** Demographic and psychological characteristics of people with multiple sclerosis (MS; n=20) and age-matched healthy individuals (HIs; n=20), with between-group comparisons^a^.

Variable and group	Values	Median (IQR)	*R* (95% CI)	*P* value
Age (y), mean (SD)				
MS	51.40 (11.02)	52.50 (45.0‐57.0)	0.20 (0.01-0.49)	.21
HI	47.30 (10.13)	49.00 (40.8‐54.2)	—^b^	—
Educational level (y), mean (SD)				
MS	13.00 (3.36)	13.00 (11.5‐15.2)	0.24 (0.02-0.51)	.12
HI	14.75 (2.20)	15.00 (13.0‐16.2)	—	—
Sex (female), n (%)				
MS	13 (65)	—	0.05^c^	.74
HI	12 (60)	—	—	—
STAI-State^d^, mean (SD)				
MS	47.35 (4.97)	47.50 (43.8‐50.0)	0.02 (0.00-0.34)	.92
HI	47.05 (4.19)	48.00 (44.0‐50.2)	—	—
STAI-Trait^e^, mean (SD)				
MS	45.50 (5.92)	47.00 (43.2‐50.0)	0.12 (0.01-0.41)	.46
HI	44.70 (4.49)	45.00 (41.0‐47.2)	—	—
I-TIPI^f^ Extraversion, mean (SD)				
MS	9.15 (2.46)	9.50 (7.0‐11.0)	0.13 (0.01-0.44)	.43
HI	8.65 (1.60)	9.00 (8.0‐10.0)	—	—
I-TIPI Agreeableness, mean (SD)				
MS	11.30 (1.98)	11.00 (10.0‐13.0)	0.17 (0.01-0.45)	.29
HI	10.65 (1.81)	10.50 (9.0‐12.0)	—	—
I-TIPI Conscientiousness, mean (SD)				
MS	10.30 (2.87)	10.00 (8.0‐13.0)	0.24 (0.02-0.54)	.13
HI	11.75 (1.48)	12.00 (11.0‐13.0)	—	—
I-TIPI Emotional Stability, mean (SD)				
MS	8.05 (3.80)	8.50 (5.0‐11.0)	0.31 (0.04-0.58)	.051
HI	10.40 (1.88)	11.00 (9.0‐12.0)	—	—
I-TIPI Openness, mean (SD)				
MS	9.30 (2.32)	9.00 (8.0‐11.0)	0.02 (0.00-0.36)	.89
HI	9.45 (2.09)	10.00 (8.0‐10.2)	—	—
PHQ-9^g^, mean (SD)				
MS	9.60 (5.78)	9.00 (5.8‐12.5)	0.70 (0.53-0.82)	<.001
HI	1.85 (2.23)	1.00 (0.0‐3.0)	—	—

a Between-group differences were assessed using Mann-Whitney *U* tests for continuous variables and *Χ*2 test for sex distribution. Effect sizes are reported as *r* (Z/√N) with 95% bootstrap CIs for continuous variables, and as Cramér *V* for sex. *P* value is reported for each comparison.

b Values refer to the MS vs HI comparison.

c This value indicates Cramér *V.*

d STAI-State: State-Trait Anxiety Inventory (State subscale).

e STAI-Trait: State-Trait Anxiety Inventory (Trait subscale).

f I-TIPI: Italian Ten-Item Personality Inventory.

g PHQ-9: 9-item Patient Health Questionnaire.

**Table 2.** Disease related and clinical information of people with multiple sclerosis (n=20).

Variable	Mean (SD; SE)
Disease duration (y)	17.65 (11.69; 2.61)
EDSS^a^	3.15 (1.92; 0.43)
DIGICOG-MS (remember and place)^b^	20.70 (4.93; 1.10)
DIGICOG-MS (listen and repeat)^c^	54.65 (9.08; 2.03)
DIGICOG-MS (generate words)^d^	26.05 (6.64; 1.49)
DIGICOG-MS (associate numbers)^e^	31.60 (10.05; 2.25)
AMSQ^f^	53.75 (28.93; 6.47)
MSIS-29^g^	69.10 (28.79; 6.44)
MFIS^h^	36.90 (24.51; 5.48)
MSNQ^i^	25.55 (13.75; 3.08)

a EDSS: Expanded Disability Status Scale.

b DIGICOG-MS (visuospatial memory test).

c DIGICOG-MS (verbal memory test).

d IGICOG-MS (semantic fluency test).

e DIGICOG-MS (information processing speed test).

f AMSQ: Arm Function in Multiple Sclerosis.

g MSIS: Multiple Sclerosis Impact Scale.

h MFIS: Modified Fatigue Impact Scale.

i MSNQ: Multiple Sclerosis Neuropsychological Questionnaire.

### Results on UX, Usability, and Human-Robot Interaction

Table 3 shows a detailed summary of results from Mann-Whitney *U* tests. The average SUS score exceeded the standard cut-off of 68 and was comparable in both groups (MS: mean 81.00, SD 12.31; HI: mean 77.75, SE 2.51, *r*=0.14; *P*=.39), indicating that participants reported a highly positive experience with Pepper.

**Table 3.** Results of statistical analyses comparing people with multiple sclerosis (MS; n=20) and age-matched healthy individuals (HIs; n=20) on technological and human-robot interaction outcomes at baseline^a^.

Variable and group	Mean (SD)	Median (IQR)	*R* (95% CI)	*P* value
SUS (%)^b^			0.14 (0.01-0.42)	.39
MS	81.00 (12.31)	81.25 (77.5‐90.0)		
HI	77.75 (11.24)	80.00 (70.0‐85.0)		
GAToRS *P*+^c^			0.17 (0.01-0.45)	.29
MS	26.55 (5.36)	27.00 (21.8‐32.0)		
HI	24.70 (4.77)	25.00 (22.0‐29.0)		
GAToRS P–^d^			0.09 (0.01-0.42)	.56
MS	11.35 (5.25)	10.50 (7.8‐13.0)		
HI	10.25 (3.78)	9.00 (8.0‐12.2)		
GAToRS S+^e^			0.05 (0.00-0.37)	.75
MS	28.25 (5.17)	28.50 (24.8‐32.5)		
HI	27.50 (4.92)	28.00 (25.0‐30.0)		
GAToRS S–^f^			0.36 (0.08-0.63)	.02
MS	25.90 (6.95)	26.50 (20.0‐32.2)		
HI	21.20 (5.55)	20.00 (16.8‐24.8)		
NASA-TLX^g^ Mental Demand			0.17 (0.01-0.47)	.29
MS	5.35 (3.74)	4.12 (2.9‐5.9)		
HI	5.86 (3.24)	5.88 (4.2‐6.6)		
NASA Physical Demand			0.03 (0.01-0.35)	.88
MS	3.17 (2.95)	2.25 (1.0‐3.8)		
HI	2.48 (1.32)	2.12 (1.5‐3.3)		
NASA Temporal Demand			0.09 (0.01-0.39)	.59
MS	3.30 (3.15)	2.50 (1.4‐3.9)		
HI	3.16 (1.81)	3.25 (1.4‐4.6)		
NASA Effort			0.14 (0.01-0.43)	.40
MS	3.83 (3.07)	2.38 (1.6‐5.2)		
HI	4.17 (2.47)	3.88 (2.4‐5.6)		
NASA Performance			0.10 (0.01-0.42)	.52
MS	16.31 (4.09)	17.12 (14.9‐19.4)		
HI	15.70 (3.79)	15.88 (13.0‐19.0)		
NASA Frustration			0.01 (0.01-0.37)	.96
MS	5.01 (5.70)	2.50 (1.0‐5.8)		
HI	3.06 (1.80)	3.00 (1.0‐4.6)		
Godspeed ANTR^h^			0.42 (0.15-0.67)	.007
MS	3.10 (0.79)	3.20 (2.6‐3.6)		
HI	2.38 (0.79)	2.40 (1.9‐2.8)		
Godspeed ANIM^i^			0.28 (0.03-0.57)	.08
MS	3.33 (0.57)	3.40 (3.0‐3.6)		
HI	2.98 (0.82)	2.80 (2.4‐3.5)		
Godspeed LIKE^j^			0.28 (0.03-0.55)	.07
MS	4.49 (0.62)	4.80 (4.2‐5.0)		
HI	4.15 (0.65)	4.10 (4.0‐4.6)		
Godspeed INT^k^			0.37 (0.09-0.61)	.02
MS	4.16 (0.69)	4.00 (3.6‐5.0)		
HI	3.49 (0.93)	3.40 (3.0‐4.2)		
Godspeed SAF^l^			0.05 (0.01-0.36)	.77
MS	2.68 (0.58)	2.50 (2.3‐3.0)		
HI	2.68 (0.69)	2.50 (2.3‐3.0)		

a Between-group differences were assessed using Mann-Whitney *U* tests. Effect sizes are reported as *r* (Z/√N) with 95% bootstrap CIs. *P* value is reported for each comparison.

b SUS: System Usability Scale.

c GAToRS *P*+: General Attitudes Towards Robots Scale—Personal Positive subscale.

d GAToRS P–: General Attitudes Towards Robots Scale—Personal Negative subscale.

e GAToRS S+: General Attitudes Towards Robots Scale—Social Positive subscale.

f GAToRS S–: General Attitudes Towards Robots Scale—Social Negative subscale.

g NASA-TLX: National Aeronautics and Space Administration Task Load Index.

h Godspeed (Anthropomorphism).

i Godspeed (Animacy).

j Godspeed (Likeability).

k Godspeed (Perceived Intelligence).

l Godspeed (Perceived Safety).

As shown by the score obtained at GAToRS S– (General Attitudes Towards Robots Scale—Social Negative subscale), people with MS perceived the introduction of robots into daily life at a community level with greater apprehension than HIs, with more focusing on the potential risks that SAR could pose at a societal level (MS: mean 25.90, SD 6.95; HI: mean 21.20, SD 5.5, *r*=0.36; *P*=.02).

Considering the mental and physical workload measured by the NASA-TLX, no differences were found between the groups (all *P* values >.05), suggesting that the proposed tasks were feasible and not excessively demanding. On the Godspeed scale, differences in ANTR and INT subscales were found. People with MS tended to perceive the robot as more anthropomorphic (natural, human-like, realistic, and fluid) than HIs (MS: mean 3.10, SD 0.79; HI: mean 2.38, SD 0.79, *r*=0.42; *P*=.007). Additionally, the INT subdomain differed between the groups (MS: mean 4.16, SD 0.69; HI: mean 3.49, SD 0.93*, r*=0.37; *P*=.02), suggesting that people with MS considered Pepper to be more competent, well-educated, responsible, and judicious. The results of Spearman correlation analyses between ANTR, INT, and psychological and clinical variables showed that ANTR was strongly associated with visuospatial memory (DIGICOG-MS: ρ=–0.632, 95% CI –0.84 to –0.26; *P*=.003), relatively strong with both verbal memory (DIGICOG-MS: ρ=–0.504, 95% CI [–0.77 to –0.08]; *P*=.02), and moderate with Agreeableness (I-TIPI: ρ=0.491, 95% CI 0.06-0.77; *P*=.03). Besides the correlation between ANTR and INT (ρ=0.645, *P*=.002, 95% CI 0.29-0.85), no other meaningful associations were observed (*Ps*>.05). See Table S1 in Multimedia Appendix 1 for the full results of the correlation analyses.

The first exploratory stepwise multiple linear regression on psychological factors was conducted to predict SUS scores. Conscientiousness (I-TIPI), negative personal attitudes toward robots (General Attitudes Towards Robots Scale—Personal Negative subscale [GAToRS P–]), and perceived upper-limb difficulty (AMSQ) explained 64% of the variance in usability (*R*²=0.637, adjusted *R*²=0.568; *F*_3,16_=9.342; *P*=.001). As shown in Table 4, higher conscientiousness was associated with greater perceived usability, whereas lower negative personal attitudes toward robots correlated with higher perceived usability—a finding consistent with prior literature suggesting that people with more open and positive dispositions toward technology tend to rate it as more usable and accessible. In addition, people with MS with higher perceived difficulties in upper-limb function reported greater usability. The second exploratory stepwise multiple linear regression on technological/robotic factors revealed that the final model with task-related frustration from NASA-TLX was retained as the sole predictor. Higher frustration was associated with lower perceived usability (*B*=−1.497, 95% CI −2.276 to −0.719, SE 0.367, β=−.693; *t*_₁₈_=−4.077; *P*=.001). The model was significant (*F*_₁,₁₈_=16.620; P=.001) and explained 48% of the variance in SUS scores (*R*²=0.480, adjusted *R*²=0.451), suggesting that, among people with MS, lower levels of task-related frustration were associated with higher perceived usability of the robot. Visual inspection of P-P plots and residual scatterplots supported the assumptions of normality and homoscedasticity in both models. Influential-case diagnostics were conducted for both regression models using Cook distance, with a threshold of 4/N=0.20. In the first model, 9 cases exceeded this threshold (Cook *D* range: 0.00‐0.72, mean 0.126, SD 0.196), and in the second model, 2 cases exceeded the threshold (Cook *D* range: 0.00‐0.365), confirming that both models should be interpreted with caution given the sensitivity to individual observations inherent in small samples. No multicollinearity was detected (model 1: VIF range 1.005‐1.022, tolerance range 0.979‐0.995; Model 2: VIF=1.000, tolerance=1.000). See Figure 3 for the heatmap of Spearman coefficients from regression analyses.

**Table 4.** Results of the first exploratory forward stepwise linear regression model predicting System Usability Scale (SUS) in people with multiple sclerosis (MS)^a^.

Predictors	B^b^ (95% CI)	SE	ß	*t* test (*df*)	*P* value
I-TIPI^c^-Conscientiousness	2.064 (0.679 to 3.449)	0.653	0.481	3.159 (16)	.006
AMSQ^d^	0.169 (0.033 to 0.306)	0.064	0.398	2.631 (16)	.02
GAToRS P–^e^	–0.937 (–1.694 to –0.180)	0.357	–0.400	–2.625 (16)	.02

a The final model included Conscientiousness (I-TIPI), negative personal attitudes toward robots (GAToRS P–), and perceived upper-limb difficulty (AMSQ) as predictors. *P* values and effect sizes with 95% CIs are reported for each comparison. *R*²=0.637, adjusted *R*2=0.568, *F*_3,16_=9.342; *P*=.001.

b Nonstandardized coefficient.

c I-TIPI: Italian Ten-Item Personality Inventory.

d AMSQ: Arm Function in Multiple Sclerosis.

e GAToRS P–: General Attitudes Towards Robots Scale—Personal Negative subscale.

**Figure 3.**
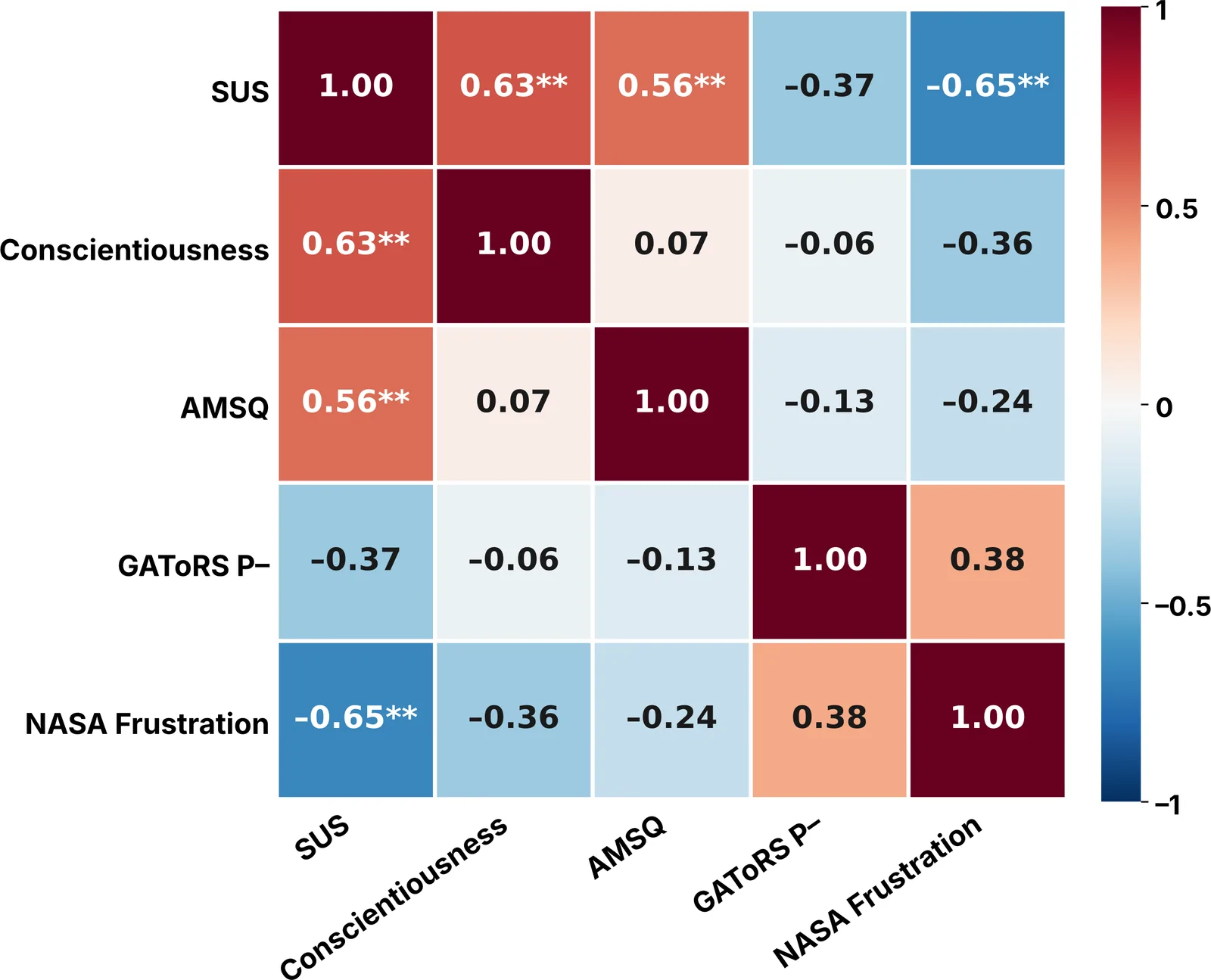
Heatmap showing Spearman correlation coefficients between meaningful predictors and System Usability Scale (SUS) scores from exploratory forward regression analyses. Color intensity represents the strength of the correlation, with darker colors indicating stronger correlations. *P* value is reported for each comparison. Positive correlations (ρ>0) are shown in red, and negative correlations (ρ<0) are shown in blue. **P<.01. AMSQ: Arm Function in Multiple Sclerosis Questionnaire; GAToRS P–: General Attitudes Towards Robots Scale—Personal Negative subscale; NASA: National Aeronautics and Space Administration.

From the exploratory NLP analysis, no meaningful differences emerged between people with MS and HIs in the extracted linguistic features (all *P* values >.05), including lexical productivity and sentiment-lexicon coverage. Within the MS group, exploratory analyses revealed meaningful associations between evaluative lexical content and psychological and user-experience measures. The number of sentiment-lexicon matches (SentN) was positively associated with SUS (*τ*=0.411; *P*=.02). After controlling for response length, residualized sentiment-lexicon coverage was positively associated with usability (*τ*=0.642; *P*<.001). This association was stable to case deletion: leave-one-participant-out Kendall’s τ values ranged from 0.592 to 0.669, while leave-two-participants-out values ranged from 0.515 to 0.715, indicating that the association was not attributable to any single participant or pair of participants. Residualized sentiment-lexicon coverage was also positively associated with GAToRS P+ (General Attitudes Towards Robots Scale—Personal Positive subscale) (*τ*=0.431; *P*=.01) and negatively associated with GAToRS P– (τ=–0.403; *P*=.02; Figure 4).

**Figure 4.**
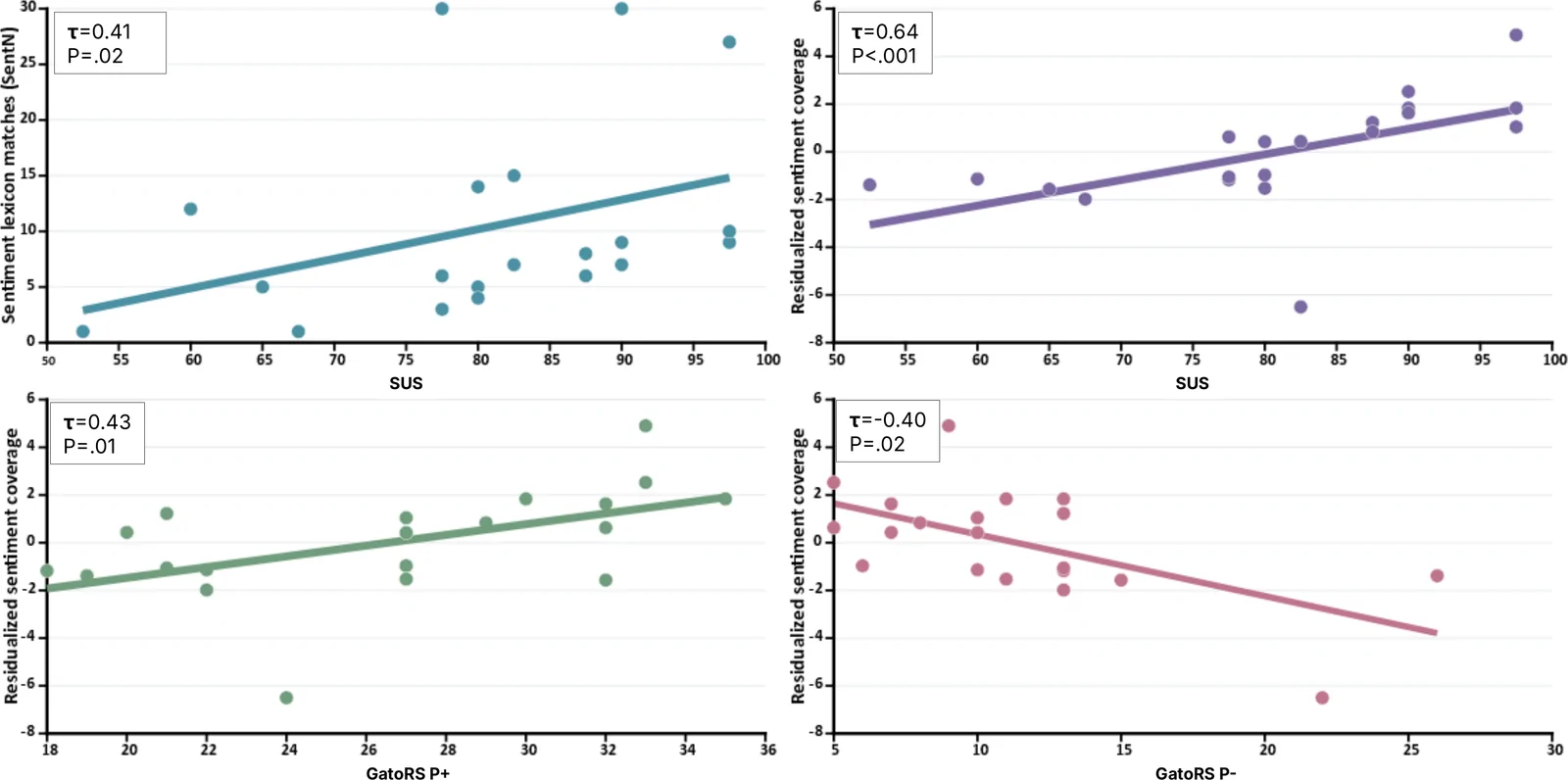
Natural language processing (NLP) associations with attitudes toward robots and perceived usability in people with multiple sclerosis. Sentiment lexicon matches and residualized sentiment coverage are reported on the *y*-axes, whereas System Usability Scale (SUS), GAToRS P+, and GAToRS P– are reported on the *x*-axes. GAToRS P–: General Attitudes Towards Robots Scale—Personal Negative subscale; GAToRS S–: General Attitudes Towards Robots Scale—Societal Negative subscale.

## Discussion

Although SAR has shown promising results in supporting everyday life, providing interaction, and facilitating cognitive and physical training in neurological and older populations, its application in MS remains largely unexplored. Our findings bridge this gap, demonstrating that Pepper was perceived as highly usable not only by HIs but also, and notably, by people with MS, a population often characterized by significant motor, cognitive, and psychological challenges. Critically, SUS scores in both groups consistently exceeded the standard cut-off of 68, and the absence of between-group differences indicates that, at least in this pilot study, MS-related challenges may not substantially hinder human-robot interaction. These preliminary findings indicate that Pepper was perceived as usable, even in the presence of MS-related challenges, and support the rationale for further investigation of SAR in MS rehabilitation contexts. These results align with Technology Acceptance Model (TAM) [47] and Unified Theory of Acceptance and Use of Technology (UTAUT) [48], 2 of the most widely used frameworks to understand why people adopt or reject new technologies, in which perceived ease of use and low effort are considered key drivers of acceptance. The high SUS scores and the absence of workload differences observed in both groups suggest that Pepper met these basic requirements, laying a favorable ground for its integration in clinical settings. Despite this high technical evaluation, meaningful differences emerged regarding social integration. People with MS expressed more cautious attitudes toward the introduction of robots in daily life, as measured by GAToRS S–, suggesting that while the robot’s functional usability is well accepted, people with MS could have greater concerns about the broader social and practical implications of SAR. This finding is consistent with studies reporting that negative attitudes and avoidance tendencies toward robots may hinder their acceptance across various societal contexts [49] and resonates with the Social Influence construct in UTAUT, which highlights how perceived community norms and collective attitudes can affect individual acceptance of new technologies. Addressing these broader social narratives around robots in health care will therefore be essential to support long-term adoption [44]. To favor a smooth introduction of SAR into human lives, it is essential to explore effective strategies targeting the reduction in negative attitudes toward robots or the fear of potential harm from their extensive use, highlighting the importance of addressing trust, transparency, and perceived risks when introducing such technologies in clinical settings.

The absence of between-groups differences in mental and physical workload, as measured by NASA-TLX, indicates that the tasks were generally manageable for both people with MS and healthy controls. This aligns with TAM and UTAUT constructs, especially with the principle that good usability relies on systems that are intuitive and efficient, minimizing the learning curve and reducing potential user frustration [50]. Surprisingly, people with MS tended to perceive the robot as more human-like (anthropomorphic) and intelligent compared to HIs, as reflected in the ANTR and INT subscales of the Godspeed questionnaire, respectively. ANTR was further associated with cognitive abilities and personality traits. Participants with lower visuospatial and verbal memory scores perceived the robot as more human-like. This pattern could be interpreted in 2 ways. First, as posited by Epley [51], anthropomorphism, as the process of attributing human-like qualities and characteristics to nonhuman agents and objects [52], may function as a cognitive compensatory strategy for individuals facing reduced processing resources [51]. Under this framework, individuals with reduced cognitive capacities may rely more heavily on heuristic social attributions, perceiving the robot as more human-like to simplify the understanding of its behavior. Second, the positive correlation between ANTR and Agreeableness from I-TIPI suggests a dispositional influence. Consistent with Epley et al [53], individual differences in anthropomorphizing nonhuman agents are often rooted in stable prosocial traits and sociality motivation—the fundamental human drive for interpersonal connection. Consequently, those who self-identify as kind, sympathetic, and cooperative may be more naturally inclined to extend these social categories to robotic partners. The finding that people with MS attributed higher levels of anthropomorphism and perceived intelligence to Pepper compared to HIs may be interpreted considering the heightened social needs commonly reported in this population. People with MS experience higher rates of social isolation and loneliness than healthy adults [16]. Building on social identity theory and research on human-robot interaction, it may be speculated that for individuals who perceive themselves as belonging to a socially marginalized group, such as people living with a chronic condition like MS, anthropomorphism may not merely humanize the robot, but symbolically reposition it as an ingroup partner, potentially attenuating intergroup boundaries between human and artificial agents [49,50]. While speculative, this interpretation is consistent with evidence that social needs and group membership influence the attribution of human-like qualities to nonhuman agents [53]. Understanding these psychological factors is crucial for tailoring interventions, improving user engagement, and optimizing the effectiveness of robot-assisted in MS.

Exploratory multiple linear regression analyses revealed that Conscientiousness (I-TIPI), personal negative attitudes toward robots (GAToRS P–), and upper-limb difficulties (AMSQ) were associated with perceived usability. Specifically, higher levels of Conscientiousness and less negative personal predispositions toward robots were associated with enhanced usability ratings. These findings also resonate with TAM and UTAUT constructs. Conscientiousness, which reflects a tendency toward goal-directed and organized behavior, may foster a more positive performance expectancy, another key driver of technology acceptance according to UTAUT. Similarly, negative personal attitudes toward robots closely mirror the concept of Behavioral Intention in TAM—that is, the degree to which an individual is predisposed toward a technology influences how they ultimately experience it. Furthermore, increased perceived difficulty in upper-limb function positively predicted usability. This suggests that individuals facing greater motor limitations may perceive assistive technologies as more indispensable, leading to a more favorable evaluation of the social robot’s use. This finding aligns with prior research indicating that participants with significant challenges in activities of daily living express a stronger preference for robotic support [54]. Finally, lower task-related frustration, as measured by the NASA-TLX, was associated with higher usability, suggesting that people with MS who experienced the interaction as less frustrating tended to evaluate the robot more favorably.

Overall, these findings highlight that human-robot interaction in MS is not uniform; psychological, prior attitudes, and functional abilities which jointly influence how people with MS perceive and interact with social robots should be considered when developing personalized rehabilitation approaches. Understanding these factors is crucial for tailoring interventions, improving user engagement, and optimizing the effectiveness of robot-assisted rehabilitation in MS. Exploratory NLP analyses revealed no between-group differences in the lexical measures examined. Within the MS group, however, associations emerged between evaluative lexical content and UX measures: participants whose written responses contained a higher density of sentiment-lexicon matches relative to response length tended to report higher usability scores and more favorable attitudes toward robots. These findings suggest that free-text language may provide information complementary to structured UX measures, although this interpretation should remain cautious given the exploratory nature of the analysis, the small sample, and the brevity of the written responses. In particular, sentiment-lexicon coverage reflects evaluative lexical density rather than positive emotional valence and should not be interpreted as evidence of a positive emotional tone. Future work using longer spoken or written responses may help determine whether linguistic signals can provide reliable complementary indicators of user experience in clinical populations. Such studies could also investigate whether richer language measures may contribute to the development of more responsive and user-centered human-robot interactions and may help clarify the role of linguistic signals as complementary indicators of UX in clinical populations. The results from NLP are further in line with key recommendations in the literature about the importance of implementing empathy-driven design in SAR, particularly ensuring that social robots can recognize and respond to human emotions through language and behavior, as discussed by Park and Whang [55]. SAR must move beyond basic functionality to provide meaningful and personalized interactions. This is confirmed by studies in autism spectrum disorders, dementia, and psychiatric disorders that emphasized that the effectiveness of SAR is linked to its ability to adapt to users’ specific needs and behavioral cues.

To our knowledge, this study represents the first preliminary exploration into the usability of social robots specifically within the MS population. By identifying the multifaceted predictors of perceived usability, spanning cognitive, personality, and clinical domains, this work marks a foundational step in understanding how these individuals perceive and evaluate SAR. These insights are instrumental for the design of tailored, ethically grounded, and user-accepted interventions, ensuring that robot-assisted rehabilitation is optimized for the unique needs of those living with MS. SAR can offer real-time feedback, maintain patient engagement, and reduce the need for continuous human supervision, thereby improving the scalability and efficiency of rehabilitation programs [13,56]. In principle, given the observed high appreciation toward robots, SAR could be strategically used in MS care as motivational agents, real-time feedback providers, or digital companions that help sustain adherence and engagement. From a rehabilitation perspective, these preliminary findings provide an initial indication that social robots may warrant further exploration as complementary tools in MS rehabilitation, though firm conclusions about their role and implementation require replication in larger and more representative samples. Addressing concerns identified by GAToRS, including perceived social risks and apprehension toward robotic integration, will be essential to foster acceptance and long-term adoption of social robots in rehabilitation contexts.

Several limitations should be acknowledged. In the absence of prior studies about the use of SAR in the MS population, no formal sample size calculation was available to conduct a formal a priori power analysis. The limited sample size, while consistent with pilot SAR studies in neurological and older populations [39,40], may reduce the statistical power to detect small effects, limiting the generalizability of the findings and precluding causal interpretations. Future studies should replicate these findings in larger samples to confirm and extend the present results. Additionally, the study focused on a limited set of tasks, and the robot did not capture other potentially informative measures of human interaction, such as gaze, number of touches, or facial expressions, due to privacy considerations. This issue is in line with Song and Luximon [56] that discussed that ethical considerations around privacy and consent in the context of human-robot interaction should be carefully managed to ensure these technologies are used responsibly and effectively. As SAR becomes more advanced, understanding how they can be seamlessly integrated into clinical workflows is crucial.

The NLP approach was selected to provide a transparent and replicable method for extracting evaluative features from a small corpus, with the explicit aim of capturing dimensions of UX not fully covered by structured questionnaires. Given the limited number of participants and the brevity of the written answers, a full qualitative content analysis was beyond the scope of the present work. Moreover, NLP results may have been influenced by response modalities: recording oral responses could have facilitated participants’ performance while reducing fatigue, particularly among people with MS. The absence of between-group differences may also reflect the limited sensitivity of lexical metrics when applied to very short texts. Future studies with larger samples and richer verbal or written data should consider complementing NLP approaches with systematic qualitative or content analysis methods to provide a more comprehensive picture of participants’ subjective experience with social robots. In this study, verbal interaction between Pepper and participants was limited and restricted to instructions. However, as indicated by [57], even minimal speech is generally well received, suggesting that the mere presence of verbal communication can enhance engagement.

## Supplementary material

10.2196/94068 Multimedia Appendix 1 Spearman correlation between Anthropomorphism and Perceived Intelligence (Godspeed subscales) and psychological variables in the multiple sclerosis group.

## Abbreviations

AISM: Italian Multiple Sclerosis Society
AMSQ: Arm Function in Multiple Sclerosis Questionnaire
ANTR: Anthropomorphism (Godspeed subscale)
EDSS: Expanded Disability Status Scale
GAToRS: General Attitudes Towards Robots Scale
GAToRS P+: General Attitudes Towards Robots Scale—Personal Positive subscale
GAToRS P–: General Attitudes Towards Robots Scale—Personal Negative subscale
GAToRS S–: General Attitudes Towards Robots Scale—Societal Negative subscale
HI: healthy individual
I-TIPI: Italian Ten Item Personality Inventory
INT: Perceived Intelligence (Godspeed subscale)
MFIS: Modified Fatigue Impact Scale
MS: multiple sclerosis
MSIS-29: 29-item Multiple Sclerosis Impact Scale
MSNQ: Multiple Sclerosis Neuropsychological Questionnaire
NASA-TLX: National Aeronautics and Space Administration Task Load Index
NLP: natural language processing
PHQ-9: 9-item Patient Health Questionnaire
PRO: patient-reported outcome
SAR: socially assistive robotics
SARA: Socially Assistive Robotic Solution for Ambient
STAI: State-Trait Anxiety Inventory
SUS: System Usability Scale
TAM: Technology Acceptance Model
UTAUT: Unified Theory of Acceptance and Use of Technology
UX: user experience
VIF: variance inflation factor
