# Supplementary material for: Usability and Acceptance of a Social Robot in People With Multiple Sclerosis: Comparative Cross-Sectional Study
Source: JMIR Rehabil Assist Technol. 2026 Oct 21;13:e94068. doi: 10.2196/94068 (PMC13686614; doi:10.2196/94068)
Supplement: Supplementary file 1 [file rehab-v13-e94068-s001.docx]

**Multimedia Appendix**

**Table S1. Spearman’s correlation between ANTR and INT from Godspeed and psychological variables in MS group.**

|  | | **Godspeed ANTR-tot** | **Godspeed INTP-TOT** |
| --- | --- | --- | --- |
| **Godspeed ANTR ^a^** | R | 1.000 | .645 |
|  | *P* | - | .002 |
| **Godspeed INT ^b^** | R | .645 | 1.000 |
|  | *P* | *.002* | - |
| **DIGICOG-MS (Remember and place) ^c^** | R | -.632 | -.429 |
|  | *P* | *.003* | .059 |
| **DIGICOG-MS (Listen and repeat) ^d^** | R | -.504 | -.289 |
|  | *P* | *.023* | .217 |
| **DIGICOG-MS (Generate words) ^e^** | R | -.168 | -.151 |
|  | *P* | .478 | .526 |
| **DIGICOG-MS (Associate numbers) ^f^** | R | -.292 | .011 |
|  | *P* | .211 | .964 |
| **STAI-STATE_TOT ^g^** | R | -.021 | .384 |
|  | *P* | .930 | .095 |
| **STAI-TRAIT_TOT ^h^** | R | -.388 | -.242 |
|  | *P* | .091 | .304 |
| **I-TIPI-EXTRAVERSION ^i^** | R | .437 | .421 |
|  | *P* | .054 | .064 |
| **I-TIPI-AGREEABLENESS** | R | .491 | .195 |
|  | *P* | *.028* | .411 |
| **I-TIPI-CONSCIENTIOUSNESS** | R | -.008 | .122 |
|  | *P* | .974 | .607 |
| **I-TIPI-EMOTIONAL STABILITY** | R | -.134 | .111 |
|  | *P* | .573 | .642 |
| **I-TIPI-OPENNES TO EXPERIENCE** | R | -.266 | -.263 |
|  | *P* | .257 | .263 |
| **PHQ-9 ^l^** | R | .115 | .048 |
|  | *P* | .628 | .842 |
| **MSNQ-TOT ^m^** | R | .349 | .015 |
|  | *P* | .132 | .951 |
| **AMSQ-TOT ^n^** | R | .223 | .075 |
|  | *P* | .345 | .752 |
| **MSIS-29-TOT ^o^** | R | .258 | .030 |
|  | *P* | .273 | .902 |
| **MFIS_FIS ^p^** | R | .366 | .192 |
|  | *P* | .112 | .417 |
| **MFIS_COG ^q^** | R | .437 | .107 |
|  | *P* | .054 | .653 |
| **MFIS_PSI ^r^** | R | .201 | -.064 |
|  | *P* | .396 | .787 |
| **MFIS_TOT ^s^** | R | .417 | .154 |
|  | *P* | .067 | .517 |

^a Godspeed (Anthropomorphism)^

^b Godspeed (Perceived Intelligence)^

^c DIGICOG-MS (Visuospatial memory test)^

^d DIGICOG-MS (Verbal memory test)^

^e DIGICOG-MS (Semantic fluency test)^

^f DIGICOG-MS (Information processing speed test)^

^g State-Trait Anxiety Inventory (State subscale)^
^h State-Trait Anxiety Inventory (Trait subscale)^

^i Italian Ten Item Personality Inventory^

^l Patient Health Questionnaire^

^m Multiple Sclerosis Neuropsychological Questionnaire^

^n Arm Function in Multiple Sclerosis^

^o Multiple Sclerosis Impact Scale^

^p Modified Fatigue Impact Scale (Physical subscale)^

^q Modified Fatigue Impact Scale (Cognitive subscale)^

^r Modified Fatigue Impact Scale (Psychosocial subscale)^

^s Modified Fatigue Impact Scale (Tot score)^
